# Identification of hub genes in rheumatoid arthritis through an integrated bioinformatics approach

**DOI:** 10.1186/s13018-021-02583-3

**Published:** 2021-07-16

**Authors:** Rui Wu, Li Long, Qiao Zhou, Jiang Su, Wei Su, Jing Zhu

**Affiliations:** 1 Department of Rheumatology and Immunology, Sichuan Provincial People’s Hospital, University of Electronic Science and Technology of China, 32 West of First Ring Road, Chengdu, Sichuan 610072 P.R. China; 2 Chinese Academy of Sciences Sichuan Translational Medicine Research Hospital, Chengdu, 610072 China

**Keywords:** Rheumatoid arthritis, Bioinformatics, Differentially expressed genes

## Abstract

**Background:**

Rheumatoid arthritis (RA) is a common chronic autoimmune disease characterized by inflammation of the synovial membrane. However, the etiology and underlying molecular events of RA are unclear. Here, we applied bioinformatics analysis to identify the key genes involved in RA.

**Methods:**

GSE77298 was downloaded from the Gene Expression Omnibus (GEO) database. We used the R software screen the differentially expressed genes (DEGs). Gene ontology enrichment analysis and Kyoto Encyclopedia of Genes and Genomes pathway were analyzed by using the DAVID online tool. The STRING database was used to analyze the interaction of differentially encoded proteins. PPI interaction network was divided into subnetworks using MCODE algorithm and was analyzed using Cytoscape. Gene set enrichment analysis (GSEA) was performed to identify relevant biological functions. qRT-PCR analysis was also performed to verify the expression of identified hub DEGs.

**Results:**

A total of 4062 differentially expressed genes were selected, including 1847 upregulated genes and 2215 downregulated genes. In the biological process, DEGs were mainly concentrated in the fields of muscle filament sliding, muscle contraction, intracellular signal transduction, cardiac muscle contraction, signal transduction, and skeletal muscle tissue development. In the cellular components, DEGs were mainly concentrated in the parts of cytosol, Z disk, membrane, extracellular exosome, mitochondrion, and M band. In molecular functions, DEGs were mainly concentrated in protein binding, structural constituent of muscle, actin binding, and actin filament binding. KEGG pathway analysis shows that DEGs mainly focuses on pathways about lysosome, Wnt/β-catenin signaling pathway, and NF-κB signaling pathway. CXCR3, GNB4, and CXCL16 were identified as the core genes that involved in the progression of RA. By qRT-PCR analysis, we found that CXCR3, GNB4, and CXCL16 were significantly upregulated in RA tissue as compared to healthy controls.

**Conclusion:**

In conclusion, DEGs and hub genes identified in the present study help us understand the molecular mechanisms underlying the progression of RA, and provide candidate targets for diagnosis and treatment of RA.

## Background

Rheumatoid arthritis (RA) is an autoimmune disease characterized by chronic inflammation, hyperproliferation of synovial tissue, and progressive destruction of multiple joints [1, 2]. RA mainly targets the synovium of diarthrodial joints [3, 4]. According to statistics, the prevalence of RA in China is about 0.5-1%, 0.5-5 new cases per 1000 people per year. RA has become one of the most common causes of disability in patients [5]. In RA, females are three times more affected than men [6]. RA manifests as osteoporosis around the joints, stenosis of the joint space of the knee joint, and bone cystic degeneration [7, 8]. The pathogenesis of RA mainly focuses on autoantibodies and immune complexes [9]. RA involves T cell-mediated antigen-specific response, T cell-independent cytokine network, and aggressive tumor-like behavior of rheumatoid synovium [10]. The initial characteristics of the membrane are abnormal growth, infiltration of inflammatory cells (macrophages, T and B lymphocytes, plasma cells, and neutrophils), and the formation of pannus [11]. Significant thickening of the synovium is the most typical pathological change of RA [12]. Studies have shown that synovial inflammation plays an important role in the pathogenesis of RA. But the exact pathogenesis of RA is unclear.

Chip technology has improved the ability to study disease pathogenesis and is an important technology for functional genomics research [13]. In recent years, with the commercialization of chips based on high-throughput platforms, this technology has gradually been used to explore disease epigenetics and screen effective biomarkers for disease diagnosis and prognosis [14]. In the expression monitoring of RA, the chip is mainly used to detect the gene expression profile of peripheral blood cells, miRNA expression, and circRNA expression.

With the development of next-generation sequencing technologies and the improvement of biological database, using bioinformatics methods to explore the relevant mechanisms is significant.

## Methods

### Microarray studies and datasets from Gene Expression Omnibus (GEO)

The microarray datasets including GSE77298 was downloaded from the GEO database (https://www.ncbi.nlm.nih.gov/geo/) using “rheumatoid arthritis” as the keyword. The microarray dataset GSE77298 from GPL570 platform contains 7 samples of RA (end-stage RA synovial biopsies) and 16 healthy controls (synovial biopsies from individuals without a joint disease). The expression micro-array datasets was Affymetrix Human Genome U133 Plus 2.0 Array.

### Differential genes expression analysis

Statistical programming language R (version 4.0.2) was used for log2 transformation of the data, and the two datasets were merged [15]. “SVA” package was used for batch correction. Differential expressed genes (DEGs) were defined as log |FC| > 0.5, and the corrected *p* < 0.05. Log |FC| > 0 means that the DEG is upregulated in RA.

### Functional annotation and pathway analysis of DEGs

DEGs were inputted into David 6.8 online tools (https://david.ncifcrf.gov/) to perform Gene Ontology (GO) analysis and Kyoto Encyclopedia of Genes and Genomes (KEGG) pathway enrichment [16, 17]. *P* < 0.05 and the gene counts > 3 were considered statistically significant.

### Protein-protein interaction (PPI) network and key genes acquisition

Using the Search Tool for the Retrieval of Interacting Genes (STRING, version 11.0, https://string-db.org/) database to analyze the PPI of proteins encoded by DEGs (medium confidence = 0.04) [18]. Cytoscape software (version 3.8.0) was used to perform visualization of PPI network. We used cytohubba plug-in to analyze the nodes of the genes with topological analysis methods, filtering with degree and stress and obtaining the key genes from the intersection of the first 15 genes sorted out in degree and stress methods respectively [19].

### Gene set enrichment analysis (GSEA)

Further GSEA was carried out for all genes that were detected by use of GSEA software (version 4.0.0), providing us another option to screen out significant differential biological functions derived after bariatric surgery [20]. The gene set arrangement was performed 1000 times per analysis. Gene sets was considered to be significantly enriched with an alpha or *P* value < 5% and a false discovery rate (FDR) < 25%.

### Quantitative real-time polymerase chain reaction (qRT-PCR)

RA was diagnosed based on the revised RA classification criteria by the American College of Rheumatology. For non-RA control, the synovial tissue samples were collected from 30 patients who underwent emergency trauma amputation. Synovial tissue was obtained and stored in liquid nitrogen and kept at −80 °C. Extraction of total RNA from tissues and cell lines was used Trizol reagent (Thermo Fisher Scientific, Waltham, USA) [21, 22]. Reverse transcription of mRNA was performed using PrimeScript RT reagent kit (TaKara, Dalian, China). The qRT-PCR experiment accorded to instructions of a SYBR Premix Ex Taq Kit (TaKaRa, Dalian, China) and performed on an ABI 7500 (Applied Biosystem). Primers used in the qRT-PCR analysis were as follows: CXCR3 forward: 5′-GAAGGTAGGCTGACAGGAAGATGAAGGG-3′, CXCR3 reverse: 5′-GAACTCGAGACCCCATAAGGAACCCAAACT-3′; GNB4 forward: 5′-ATGCCTTGCACTGAAAGAAG-3′, GNB4 reverse: 5′-ATACGGCTACGCCCTTCTTG-3′; GAPDH Forward: 5′-CACGAATUATTCAACGGTCGATCAAGG-3′, GAPDH reverse: 5′-GTTCACACCCATCACAAACATGG-3′; CXCL16 forward: 5′-CTTTCATCGATAGCGCA-3′, CXCL16 reverse: 5′-AACGCTTCACGAATTTGCGT-3′. A ratio relative to the GAPDH was used as internal control.

### Statistical analysis

Each experiment was performed at least three times. All data were expressed as mean ± standard deviation (SD). Statistical analyses were determined by Student’s t test, and the differences between two groups or more than two groups were detected using ANOVA. A *p* value of less than 0.05 was considered statistically significant.

## Results

### Hierarchical clustering for sample selection

The total samples were analyzed by hierarchical clustering, no samples were with high heterogeneity and eliminated. Finally, 23 samples were included for analysis.

### Identification of DEGs

**Fig. 1:**
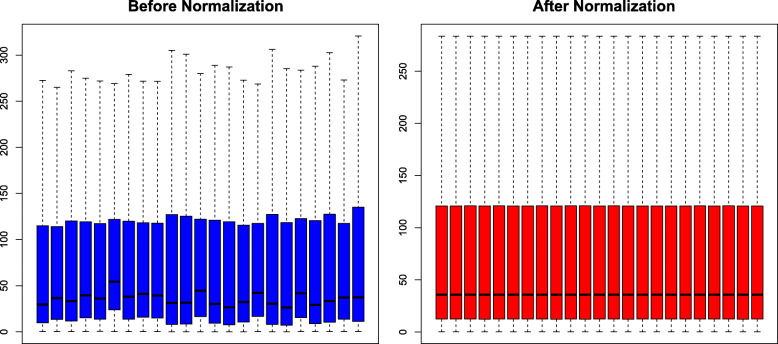
Comparison of expression value between before normalization and after normalization

The blue bar represents the data before normalization, and the red bar represents the normalized data. After normalization, Fig. 1 depicts that the log2 ratios in the three pairs of samples are almost identical.

**Fig. 2:**
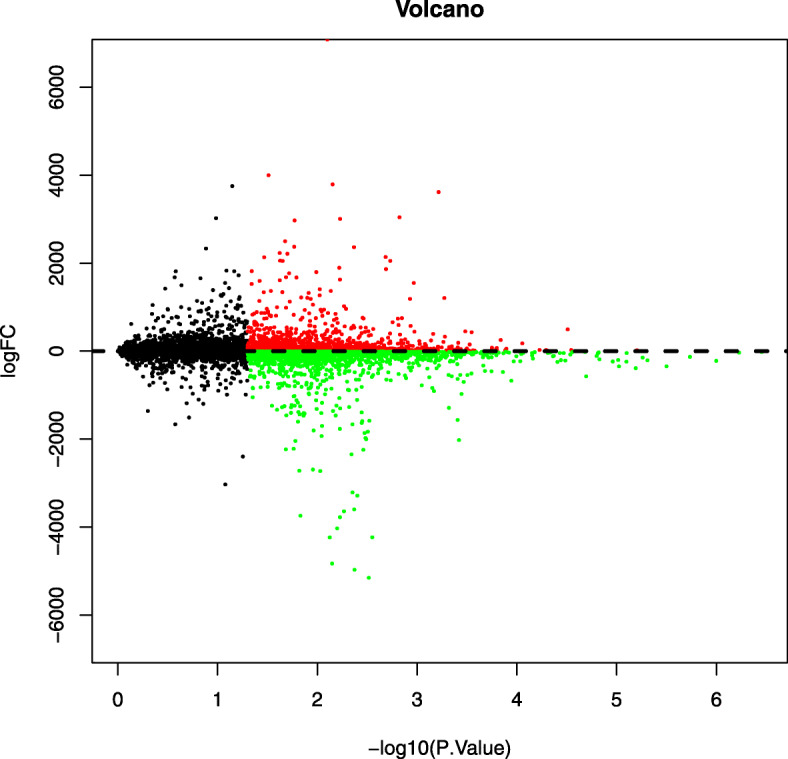
Volcano plot of the differentially expressed genes; (red) upregulated genes; (green) downregulated genes; and (black) non-differentially expressed genes

**Fig. 3:**
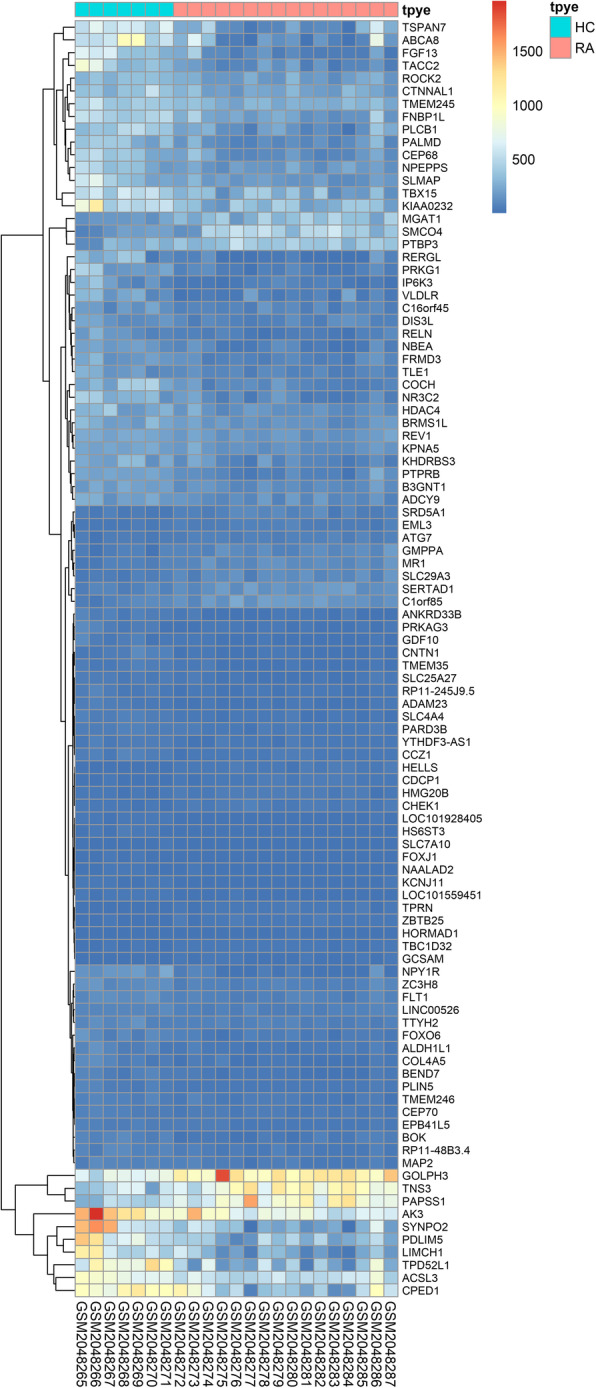
Heat map of the standardized expression of the top 100 differentially expressed genes across the 23 samples

A total of 4062 DEGs were screened out, including 1847 high expression genes and 2215 low expression genes. R was used to make results visualization and draw volcano map (Fig. 2) and heat map (Fig. 3).

### GO and KEGG enrichment analysis of DEGs

**Fig. 4:**
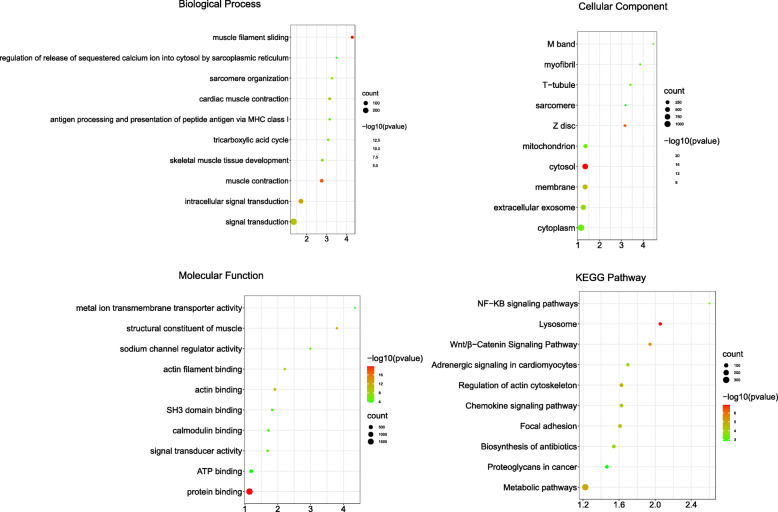
Gene Ontology (GO) and KEGG pathway enrichment analyses of DEGs

In GO analysis, DEGs were divided into three categories: biological process, cellular component, and molecular function. In the biological process, DEGs were mainly concentrated in the fields of muscle filament sliding, muscle contraction, intracellular signal transduction, cardiac muscle contraction, signal transduction, skeletal muscle tissue development, sarcomere organization, antigen processing and presentation of peptide antigen via MHC class I, tricarboxylic acid cycle, and regulation of release of sequestered calcium ion into cytosol by sarcoplasmic reticulum (Fig. 4).

In the cellular components, DEGs were mainly concentrated in the parts of cytosol, Z disk, membrane, extracellular exosome, mitochondrion, M band, cytoplasm, T-tubule, myofibril, sarcomere and so on (Fig. 4).

In molecular functions, DEGs were mainly concentrated in protein binding, structural constituent of muscle, actin binding, actin filament binding, signal transducer activity, calmodulin binding, sodium channel regulator activity, SH3 domain binding, metal ion transmembrane transporter activity, and ATP binding (Fig. 4).

KEGG pathway analysis shows that DEGs mainly focuses on pathways about lysosome, Wnt/β-catenin signaling pathway, metabolic pathways, regulation of actin cytoskeleton, focal adhesion, chemokine signaling pathway, adrenergic signaling in cardiomyocytes, biosynthesis of antibiotics, NF-κB signaling pathway, and proteoglycans in cancer (Fig. 4).

### PPI network analysis of DEGs

**Fig. 5:**
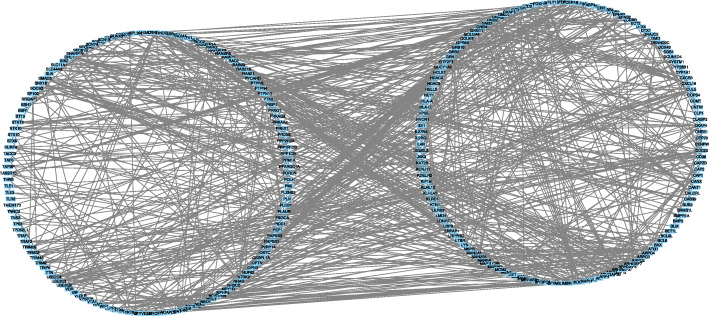
Protein-protein interaction of the differentially expressed genes between RA tissue and healthy controls

Protein-protein interaction network with a total of 198 nodes and 356 relationship pairs was obtained, and genes in protein-protein interaction, such as RNF4, CDC20, UBE2D4, and UBE2Q2, were recognized as key nodes in protein-protein interaction (Fig. 5).

**Fig. 6:**
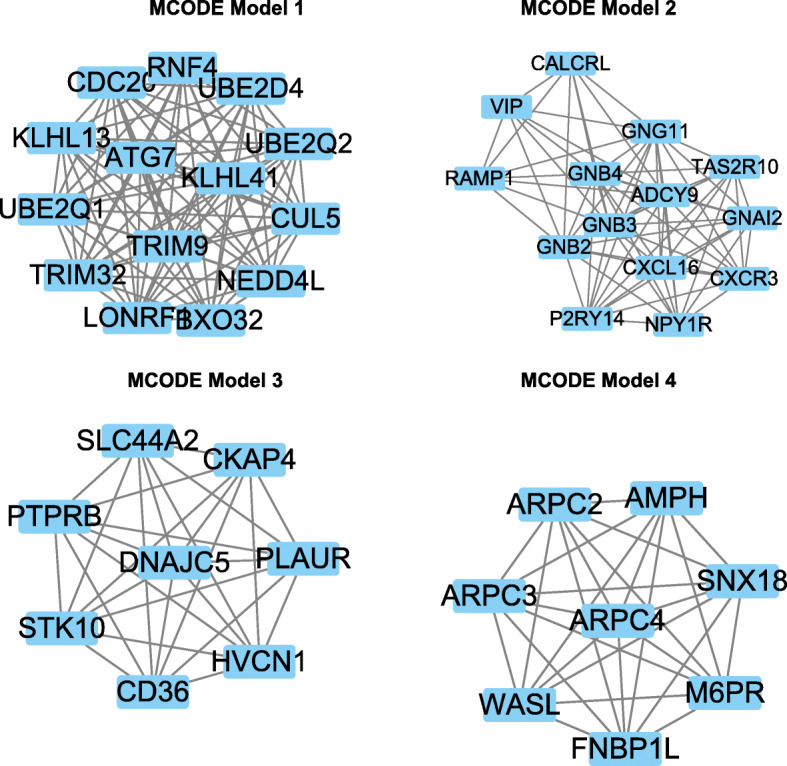
**A** Module 1. **B** Module 2. **C** Module 3. **D** Module 4. The modules are extracted using Molecular Complex Detection (MCODE) plugin of Cytoscape software

A total of 20 core genes with a degree ≥ 20 selected by MCODE were obtained from the protein-protein network, and they were considered to be candidate core genes. In MCODE model 1, key genes were as follows: RNF4, UBE2D4, UBE2Q2, CUL5, NEDD4L, BXO32, LONRF, TRIM32, UBE2Q1, KLHL13, CDC20, ATG7, KLH41, and TRIM9 (Fig. 6).

In MCODE model 2, key genes were as follows: CALCRL, GNG11, TAS2R10, GNAI2, CXCR3, NYP1R, P2RY14, GNB2, RAMP1, VIP, GNB4, GNB3, CXCL16, and ADCY9.

In MCODE model 3, key genes were as follows: SLC44A2, CKAP4, PLAUR, HVCN1, CD36, STK10, PTPRB, and DNAJC5 (Fig. 6).

In MCODE model 3, key genes were as follows: ARPC2, AMPH, SNX18, M6PR, NBP1L, WASL, APRC3, and APRC4 (Fig. 6).

### GSEA

**Fig. 7:**
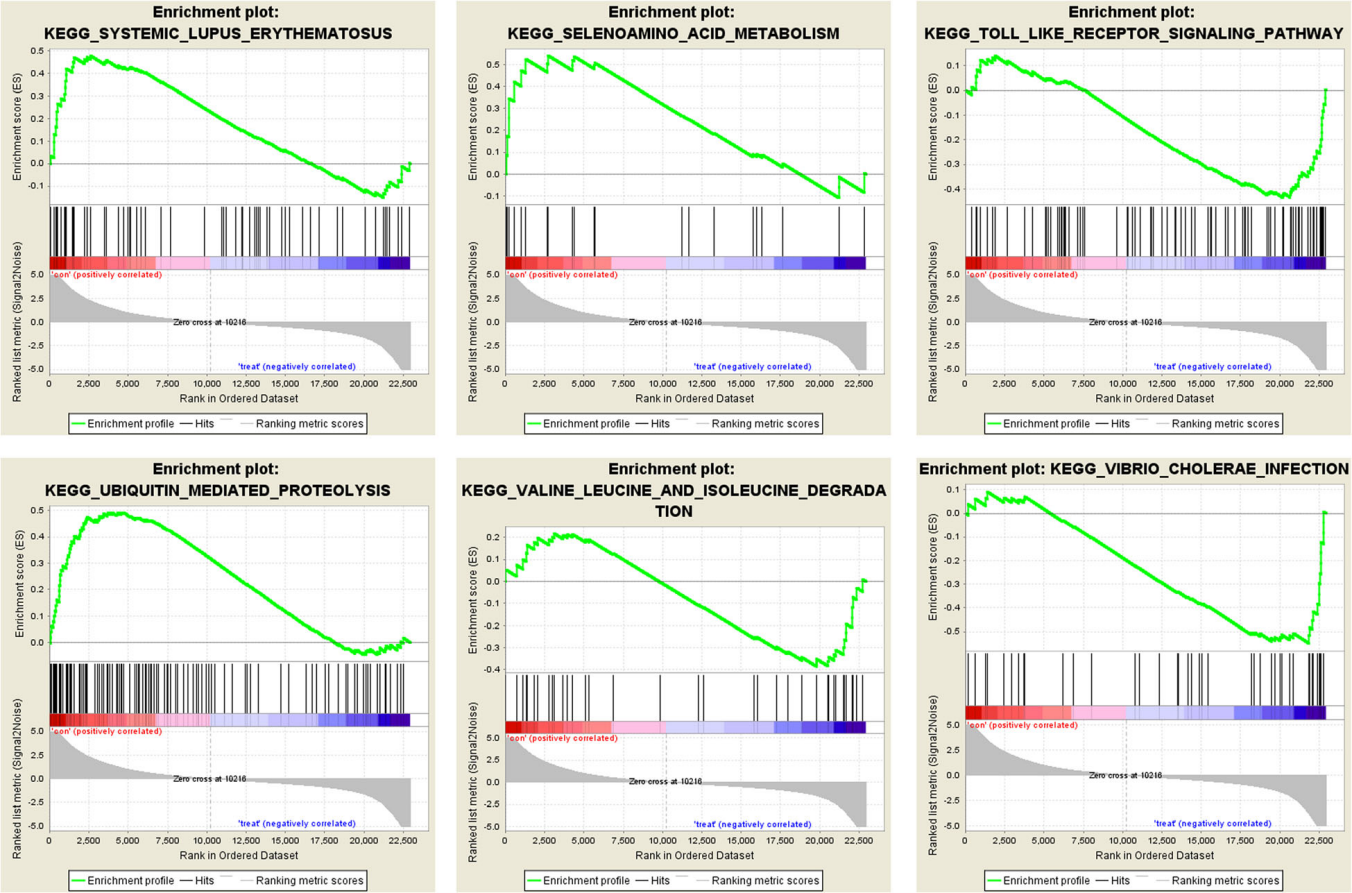
GSEA analysis of the 23 samples from RA tissues and healthy controls

The analysis indicated that the most significant-enriched gene sets included the systemic lupus erythematosus, selenoamino acid metabolism, toll-like receptor signaling pathway, ubiquitin-mediated proteolysis, valine leucine and isoleucine degradation, and cholerae infection (Fig. 7).

### PCR

**Fig. 8:**
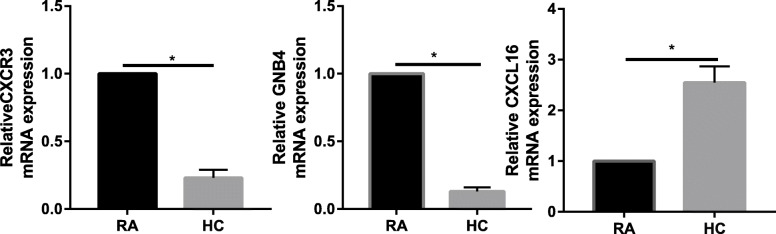
PCR results of the expression of CXCR3, GNB4, and CXCL16 between RA and healthy controls. *P < 0.05

The quantitative PCR (qPCR) results indicated that CXCR3 expression was significantly upregulated in RA synovial tissue compared with healthy control (Fig. 8).

Moreover, GNB4 was significantly upregulated in RA synovial tissue compared with healthy control (Fig. 8).

However, CXCL16 was significantly downregulated in RA synovial tissue compared with healthy control (Fig. 8).

## Discussion

In the present study, we analyzed GSE77298 microarray dataset to screen DEGs between end-stage RA synovial biopsies and 16 synovial biopsies from individuals without a joint disease.

GO and KEGG enrichment analyses were performed to explore interactions among the DEGs. CXCR3 was identified as the core gene that involved into the progression of RA. Bakheet et al. [23] found that CXCR3 antagonist AMG487 suppresses RA pathogenesis and progression by shifting the Th17/Treg cell balance. Therefore, CXCR3 antagonists could be used as a novel strategy for the treatment of inflammatory and arthritic conditions. Another core gene should be noticed is the GNB4. Previous study found that GNB4 can be a candidate diagnostic biomarker in inflammatory bowel diseases [24]. As for CXCL16, we also found that CXCL16 can be as a candidate core gene of RA according to the MCODE analysis. Li et al. [25] revealed that CXCL16 is a modulator of RA disease progression. They performed in vitro study and found that CXCL16 upregulates RANKL expression in RA synovial fibroblasts through the JAK2/STAT3 and p38/MAPK signaling pathway.

The main innate immune-related signaling pathways include NF-κB signaling pathway and TRIM32 signaling pathway. Wang et al. [26] found that the TRIM3 expression was significantly downregulated in RA patients than that of the healthy controls. Overexpression of TRIM3 promoted the p53 and p21 expression, while inhibited cyclin D1 and PCNA expression. More importantly, knockdown of TRIM3 expression could partially reversed the inhibition effects of SB203580 (p38 inhibitor) on the inhibition of cell proliferation.

Rheumatoid arthritis is an autoimmune nature joint disease with irreversible cartilage destruction and bone erosion. The DEGs were mainly enriched in muscle filament sliding, muscle contraction, intracellular signal transduction, and cardiac muscle contraction. KEGG pathway analysis shows that DEGs mainly focuses on pathways about lysosome, Wnt/β-catenin signaling pathway, metabolic pathways, regulation of actin cytoskeleton, focal adhesion, chemokine signaling pathway, adrenergic signaling in cardiomyocytes, biosynthesis of antibiotics, NF-κB signaling pathways, and proteoglycans in cancer.

Studies have shown that the development of RA may depend on the common changes in the expression of specific key genes. Xiong et al. [27] revealed that upregulated genes in RA were significantly enhanced in protein binding, the cell cytosol, organization of the extracellular matrix (ECM), regulation of RNA transcription, and cell adhesion. Shchetynsky et al. [28] revealed that ERBB2, TP53, and THOP1 were new candidate genes in the pathogenesis of RA.

KEGG pathway analysis revealed that NF-KB signaling pathway involved into the progression of OA. Xing et al. [29] revealed that miR-496/MMP10 is involved in the proliferation of IL-1β-induced fibroblast-like synoviocytes via mediating the NF-κB signaling pathway. The NF-kB signaling pathway may also have an important role in OA progression because NF-kB molecule has key role in immune response regulation. In this study, we also found that DEGs mainly enriched into the NF-KB signaling pathway.

Lysosomes are membrane-bound organelles with roles in processes involved in degrading and recycling cellular waste. In KEGG pathway enrichment analysis, we found that DEGs also enriched into the lysosomes pathway. Lysosomes can be as a therapeutic target for RA [30].

There were some limitations in our study. First, all patients had a pathological diagnosis of RA; however, correlation between DEGs and disease severity did not examine in depth. Second, though we examined the expression of the DEGs between RA and healthy controls, the potential pathway that involved into the RA was not examined. Future studies should be performed to identify the detailed pathway that participated into the progression of RA.

## Conclusions

In conclusion, DEGs and hub genes identified in the present study help us understand the molecular mechanisms underlying the progression of RA, and provide candidate targets for diagnosis and treatment of RA.

## Data Availability

The datasets used and/or analyzed during the current study are available from the corresponding author on reasonable request.

## Abbreviations

RA: Rheumatoid arthritis
GEO: Gene Expression Omnibus
DEGs: Differentially expressed genes
GSEA: Gene set enrichment analysis
GO: Gene Ontology
KEGG: Kyoto Encyclopedia of Genes and Genomes
PPI: Protein-protein interaction
STRING: Search Tool for the Retrieval of Interacting Genes
qRT-PCR: Quantitative real-time polymerase chain reaction
SD: Standard deviation
ECM: Extracellular matrix
